# Endothelial progenitor cells in cardiovascular diseases: from biomarker to therapeutic agent

**DOI:** 10.1186/2050-490X-1-9

**Published:** 2013-12-06

**Authors:** Hui-Bin Liu, Yuan-Feng Gong, Chang-Jiang Yu, Ying-Ying Sun, Xin-Yuan Li, Dan Zhao, Zhi-Ren Zhang

**Affiliations:** Departments of Clinical Pharmacy and Cardiology, the 2nd Affiliated Hospital, Harbin Medical University, The Key Laboratory of Myocardial Ischemia, Chinese Ministry of Education, Harbin, 150086 P.R. China

**Keywords:** Endothelial progenitor cells, Biomarkers, Cardiovascular regeneration

## Abstract

Regenerative medicine techniques to recover cardiac and vascular function are being increasingly investigated as management strategies for cardiovascular diseases. Circulating endothelial progenitor cells (EPCs) derived from bone marrow are immature cells capable of differentiating into mature endothelial cells and play a role in vascular reparative processes and neoangiogenesis. The potency of EPCs for cardiovascular regeneration has been demonstrated in many preclinical studies and therapeutic utility of EPCs has been evaluated in early-phase clinical trials. However, the regenerative activity and efficiency of the differentiation of EPCs are still limited, and a directed differentiation method for EPCs cells has not been fully demonstrated. In this review, we introduce the role of circulating EPCs as biomarkers of cardiovascular diseases and medical applications of EPCs for cardiovascular regeneration.

## Introduction

Circulating endothelial progenitor cells (EPCs) are bone marrow derived peripheral blood mononuclear cells that have the capacity to proliferate, migrate, and differentiate into mature endothelial cells (ECs) [1]. EPCs were first discovered in human peripheral blood [2] and were shown to incorporate into sites of physiological or pathological neovascularization [3–5]. The discovery of EPCs has greatly enhanced our understanding of blood vessel formation. Accumulated evidence has elucidated that EPCs provide a postnatal vasculogenesis mechanism for neovascularization and vascular remodeling [6, 7]. EPCs have a diverse of physiological functions and participate in the recovery processes of myocardial ischemia and infarction [8], limb ischemia [9], wound healing [10, 11], atherosclerosis [12], endogenous endothelial repair [13], and tumor vascularization [14]. Clinical trials have demonstrated that EPC therapy is safe and feasible for the treatment of cardiovascular diseases. In addition, circulating EPCs levels are considered as biomarkers for coronary and peripheral artery disease. However, despite significant steps toward defining their potential for both diagnostic and therapeutic purposes, further progress has been mired by unresolved questions regarding the definition and the mechanism of action of EPCs. This review will highlight the potential value of EPCs as the biomarkers and a potential therapeutic method for cardiovascular diseases.

## Review

### Endothelial progenitor cells (EPCs)

Adult bone marrow (BM) is a rich reservoir of tissue-specific stem and progenitor cells and EPCs constitutes 1–5 percent of the total bone marrow cells [15]. Based on the originated status, circulating EPCs can be subdivided into two main categories, hematopoietic lineage EPCs (HEPCs) and nonhematopoietic lineage EPCs (NHEPCs). The HEPCs originate from BM and identification of HEPCs is associated with the methods and markers of hematopoietic stem cells (HSCs) [16]. Nevertheless, it is still difficult to clearly distinguish between EPCs and HSCs for lacking of specific and selective markers for primary EPCs. The NHEPCs are isolated from blood or tissue samples but not BM cells, which can be successive cultured and distinguished by their rather obvious endothelial cell phenotype [17, 18]. The origin of NHEPCs remains to be clarified, but they are generally thought to be derived from nonhematopoietic tissue-prone lineage stem cells or organ blood vessels but not like the HSCs [19]. EPCs are multiple cell types capable of differentiating into the endothelial lineage but not a single cell type [14]. The cells differentiated from EPCs possess the characteristic of mature ECs, including expressions of CD31^+^, E-selectin^+^, endothelial nitric oxide synthase (eNOS)^+^, and uptake of acetylated low density lipoprotein [20]. However, it is difficult to define EPCs precisely because of a lack of consensus regarding the best EPC source, the optimal isolation and culture techniques, and the phenotypes and characteristics that are especially crucial for EPC identity.

In 1997 Asahara and colleagues published a landmark paper in Science [2], showing that EPCs in adult human peripheral blood were CD34^+^/VEGFR-2^+^ (vascular endothelial growth factor receptor 2) mononuclear cells. Subsequent studies confirmed that CD34^+^ cells from bone marrow or umbilical cord blood also had the capacity to differentiate into mature ECs *in vitro* and *in vivo* in mouse models [4, 21], thereby contributing to neoendothelialization and neovascularization in the adult organism. However, both CD34 and VEGFR-2 are expressed on mature ECs. Thus, better markers are needed. The stem cell marker CD133 (AC133) may be a more precise marker for defining EPCs, because CD133 is not expressed on mature endothelial cells [14]. However, CD133 expression declines as differentiation progresses, whereas CD34 expression is maintained, and the expression of endothelial markers (*e.g.*, VEGF-2, von Willebrand factor [vWF], eNOS) increases [22]. Consequently, marker expression has been used to distinguish between early EPCs (e.g., CD133^+^/CD34^+^ cells), early and late circulating EPCs (e.g., CD133^-^/CD34^+^ cells), and EPCs that are nearing maturity (e.g., vWF^+^ cells).

EPCs locating to damaged tissues and organs proceeding vascular regeneration do not only participate in the formation of the neovasculature but also produce a variety of proangiogenic cytokines and growth factors, promoting proliferation and migration of pre-existing ECs, activating angiogenesis to contribute to vascular regeneration [23, 24]. This paracrine aspect of EPC activity was reflected by the presence of various cytokines and other secreting pro-angiogenic factors in EPCs such as VEGF, stroma derived factor (SDF)-1α, angiopoietin-1 (Ang-1), hepatic growth factor (HGF), insulin-like growth factor (IGF)-1, and eNOS/iNOS (inducible nitric oxide synthase) [25–27]. Therefore, EPCs can mediate tissue-protective effects and contribute to neovascularization via direct vasculogenesis in ischemic tissues and indirect production of proangiogenic factors to pre-existing ECs.

### EPCs as potential biomarkers of cardiovascular diseases

Reduced numbers and impaired functionality of EPCs have been found in several clinical conditions such as diabetes mellitus [28, 29], hypertension [30–32], heart failure [33] and chronic kidney disease [34–36]. It has been shown that the peripheral EPC number is reduced while EPC function is impaired and the numbers of circulating EPCs are significantly reduced in patients with established coronary artery disease [37] and stroke [38]. However, the number of EPCs is increased in patients with an acute coronary syndrome, such as acute myocardial infarction [39] or unstable angina [40, 41], because they are mobilized from the bone marrow into the bloodstream. Importantly, the level of circulating CD34^+^/VEGFR-2^+^ EPCs further decline in the later stages of atherosclerosis in different districts, such as coronary [42–44], carotid and cerebral [45, 46], and peripheral atherosclerosis [37, 47]. Correlations were also found between severity of the atherosclerotic burden and EPC levels, [38, 46] indicating that low EPCs represent a biomarker of the systemic atherosclerotic involvement.

It has been shown that hypertension patients with coronary artery disease have reduced levels and migratory capacity of EPCs [48]. Moreover, the concentration of circulating EPCs is significantly reduced in refractory hypertension as compared to healthy subjects [49]. Imanishi et al. has reported that EPC senescence is accelerated in both experimental hypertensive rats and patients with essential hypertension, which may be related to telomerase inactivation [32, 50]. They found the hypertension-induced EPC senescence might affect the process of vascular remodeling [50]. Thereafter, Delva *et al.* reported no alteration in the number or functional activity of EPCs in 36 patients with essential hypertension [51]. With regard to pulmonary hypertension, some studies have shown there is a decrease in the levels of EPC [52–54], while others report that normal levels of EPC or an increase in EPC number [55, 56]. Therefore, at present, there is no evidence of a clear independent relationship between hypertension and the number of circulating EPCs [57].

Valgimigli *et al.* tested EPC levels in patients with heart failure (HF), and they found that EPC mobilization occurred in HF and showed a biphasic response, with elevation and depression in the early and advanced phases, respectively [58]. The increased EPCs had been shown as a reflection of a functional bone marrow response to diffuse and severe endothelial damage during the early stages of HF, but an additional and significant increase of tumor necrosis factor (TNF-α) counteracted and overwhelmed the elevation of EPC mobilization in advanced disease phases by exerting a possible suppressive effect on hemopoiesis [59]. In contrast, another study showed that EPC levels were probably not influenced by the aetiology of HF, but rather correlated with the patient’s clinical status [60]. A recent report has shown that HF patients with both preserved ejection fraction and reduced ejection fraction have significantly decreased circulating EPC levels, enhanced systemic inflammation, and higher N-terminal pro-brain natriuretic peptide levels compared to controls [61].

Although the studies of EPCs as the biomarkers in cardiovascular diseases have not generated a conclusive result, these cells have widened the spectrum of cellular biomarkers and have supported the concept that circulating EPCs may affect the cardiovascular system.

### EPCs as a therapeutic agent

Besides as potential cardiovascular risk biomarkers, EPCs have been extensively studied for their pathophysiological and therapeutic implications in cardiovascular diseases. However, several obstacles exist before large scale use of EPCs. For instance, the relatively rare cells must be expanded in sufficient numbers from peripheral blood, and possible changes in phenotype may increase the risk of cell senescence after *in vitro* enumeration of progenitor cells. Increasing the number and/or improving the function of EPCs may be promising in the treatment of atherosclerotic disease, ischemia or HF.

Myocardial ischemia caused by coronary artery disease can be attenuated by the development of collateral circulation; following the role of EPCs in neovascularization was recognized, investigators have begun to evaluate the potential therapeutic impact of EPCs. It has been shown that recovery of blood flow was greater in mice with hindlimb ischemia treated with EPCs than in control mice and in mice that received mature ECs, and that histological examinations confirmed EPC incorporation and differentiation into ECs [9]. Kawamoto and co-workers [8] evaluated EPC therapy in nude rats after acute myocardial infarction. These investigators found that intravenous administration of *ex vivo* expanded human EPCs could inhibit myocardial fibrosis and was able to preserve myocardial function. In addition, chronic treatment with bone marrow derived progenitor cells from young non-atherosclerotic apolipoprotein E knock-out (ApoE^-/-^) mice prevents atherosclerosis from progression in ApoE^-/-^ recipients [62]. In contrast, treatment with bone marrow cells from older ApoE^-/-^ mice with atherosclerosis is much less effective. These results suggest that ApoE gene deficiency may not affect EPC repairing efficiency but that the chronic stimulation of EPCs in older ApoE^-/-^ mice significantly weakens EPC repairing function. In addition, it has been demonstrated that EPC therapy improves regional systolic function accompanied by cardiac hypertrophy in porcine acute myocardial infarction models. The effect of EPCs on cardiac hypertrophy is mediated by paracrine secretion of cardiotrophic factors including TGFβ1 [63].

Several small-scale clinical trials have been performed to evaluate the use of bone marrow cell transplantation in treatment of cardiovascular diseases. However, the available clinical studies with respect to administration of circulating progenitor cells in cardiovascular diseases are mainly about CD34^+^ cells [64, 65]; only a few studies suggest the role of CD34^+^/CD133^+^ cells in cardiovascular diseases. Intracoronary infusion of CD133^+^ cells after acute myocardial infarction led to an improvement of left ventricular ejection fraction [66]. 167 patients with refractory angina received intramyocardial injections of mobilized, autologous CD34^+^ cells resulted in a significant improvement in angina frequency and a significant improvement in exercise response [64]. Another study suggests that injection of CD133^+^ cells into the myocardial border zone improves left ventricular function [67, 68]. In patients with dilated cardiomyopathy, administration of autologously transplanted CD34^+^ cells led to an improvement of left ventricular ejection fraction [65]. Data collected from *in vivo* and *in vitro* experiments suggest that blockade of C-X-C chemokine receptor type 4 is sufficient to mobilize EPCs and to increase recruitment of EPCs to the neovasculature [69]. There is growing number of studies regarding EPC therapy in cardiovascular diseases, however, this therapeutic intervention in human remains to be further validated.

Although the preclinical and clinical studies reviewed here generally give strong support to the therapeutic potential of EPCs in the treatment of cardiovascular diseases, the clinical application of EPCs is limited by several factors. At first, the relatively shortage of circulating EPCs makes it difficulty to expand sufficient number of cells for therapeutic application without inducing the risk of cell senescence and change in phenotype [2, 70]. Furthermore, the number and availability of EPCs are sensitive to some pathologic state, such as aging and diabetes which are always accompanied by cardiovascular diseases [29, 71, 72], this severely restricts the ability of autologous EPCs to treat patients with cardiovascular diseases. Finally, for a successful therapeutic EPC-based approach, it is essential to get optimal quality/quantity of EPCs, such as ameliorating EPC purification and expansion methods, improving the administration and cellular application techniques, and recovering the disease-based dysfunction and/or senescence of patient-derived EPCs.

## Conclusions

The involvement of EPCs in postnatal vasculogenesis and endothelial repair is supported by growing preclinical evidence. EPCs also participate in arteriogenesis in cardiovascular diseases. The mechanisms by which EPC-mediated vessel growth and repair in cardiovascular diseases are not fully understood, the vasculogenic effects are thought to be attributed to the variety of angiogenic factors produced by EPCs. EPC-based therapy is still in very early stage, as critical questions regarding EPC survival, timing of administration, and phase- or activity-dependent efficacy of the diseases need to be addressed. The regenerative potency of EPCs will continuously be evaluated by ongoing, randomized, controlled clinical trials.
